# A Pharmacopœia for the Treatment of Diseases of the Larynx, Pharynx and Nasal Passages

**Published:** 1885-07

**Authors:** 


**A Pharmacopœia for the Treatment of Diseases of the Larynx, Pharynx and Nasal Passages,** with remarks on the selection of remedies and choice of instruments, and in the methods of making local applications, by George M. Lefferts, A. M., M. D., Clinical Professor of Laryngoscopy and Diseases of the Throat, College of Physicians and Surgeons, N. Y. Second edition, revised and enlarged. New York and London: G. P. Putnam's Sons. 1884.

The title sufficiently indicates the scope of this little work, and the name of the author is a sufficient recommendation. It is abundantly illustrated, and the general practitioner will find it of practical worth.

